# Intra‐Islet Paracrine Regulation of Glucagon Secretion During Hypoglycemia, Euglycemia, and Hyperglycemia

**DOI:** 10.1111/nyas.70384

**Published:** 2026-09-14

**Authors:** Samaneh Fatehi, Jonathan V. Rocheleau, Michael C. Riddell

**Affiliations:** ^1^ School of Kinesiology and Health Science York University Toronto Ontario Canada; ^2^ UHN Research Institutes University Health Network Toronto Ontario Canada; ^3^ Institute of Biomedical Engineering University of Toronto Toronto Ontario Canada; ^4^ Departments of Medicine and Physiology University of Toronto Toronto Ontario Canada

**Keywords:** glucagon counterregulation, glucagon‐like peptide, hypoglycemia, insulin, islet of Langerhans, somatostatin, somatostatin receptor antagonist, type 1 diabetes, type 2 diabetes

## Abstract

The regulation of glucagon and insulin secretion, from pancreatic α‐ and β‐cells, respectively, is essential for maintaining blood glucose within the physiological set point. Insulin secretion is stimulated as glucose rises from euglycemia, with incretin hormones modulating the secretion in a glucose‐dependent manner. Glucagon secretion is elevated with hypoglycemia but also with hyperglycemia. Within the pancreatic islet, α‐cell glucagon output is regulated by complex neuroendocrine and paracrine signals from insulin‐releasing β‐cells and somatostatin‐secreting δ‐cells. This network of intra‐islet crosstalk tunes glucagon secretion in response to changes in glucose levels. In diabetes, intra‐islet paracrine crosstalk between α‐, β‐, and δ‐cells is disrupted. Autoimmune destruction of β‐cells or impaired α‐cell responsiveness to residual β‐cell signals leads to hyperglucagonemia. Conversely, the loss of controlled insulin secretion, along with elevated somatostatin levels, attenuates glucagon responses to hypoglycemia or to prolonged exercise. Antihypoglycemia therapies such as selective somatostatin receptor 2 antagonists have emerged as a novel strategy in managing iatrogenic insulin‐induced hypoglycemia. This review summarizes the latest experimental findings on the regulation of glucagon secretion by pancreatic β‐ and δ‐cells, examines how this paracrine regulation of α‐cell secretion is affected in diabetes, and highlights potential interventions aimed at restoring physiological glucagon dynamics.

## Graphical abstract

Glucagon, a counterregulatory hormone released from pancreatic α‐cells, maintains glucose homeostasis by acting as a protective barrier against hypoglycemia and an amplifier of insulin release when glucose levels rise with feeding. In the body, glucagon secretion is regulated by circulating glucose levels, with elevations in amino acids and via other inter‐ and intra‐islet paracrine factors. Loss of coordinated regulation of α‐cell release leads to aberrant glucagon secretion in diabetes characterized by counterregulatory failure during hypoglycemia and pathological elevations during hyperglycemia.

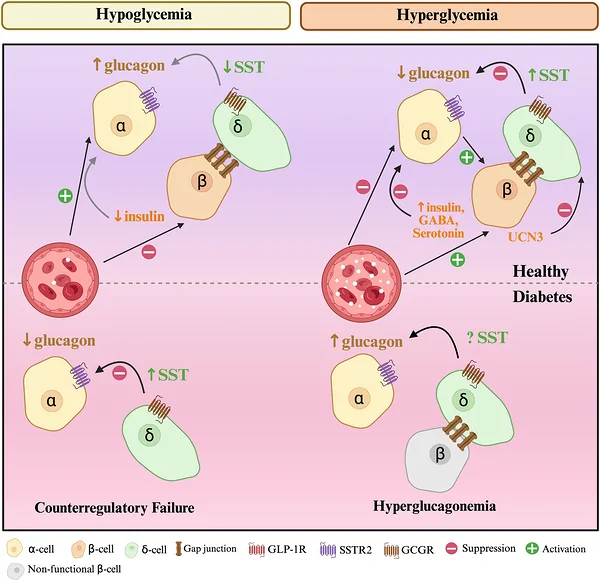

## 1 Introduction

**FIGURE 1:**
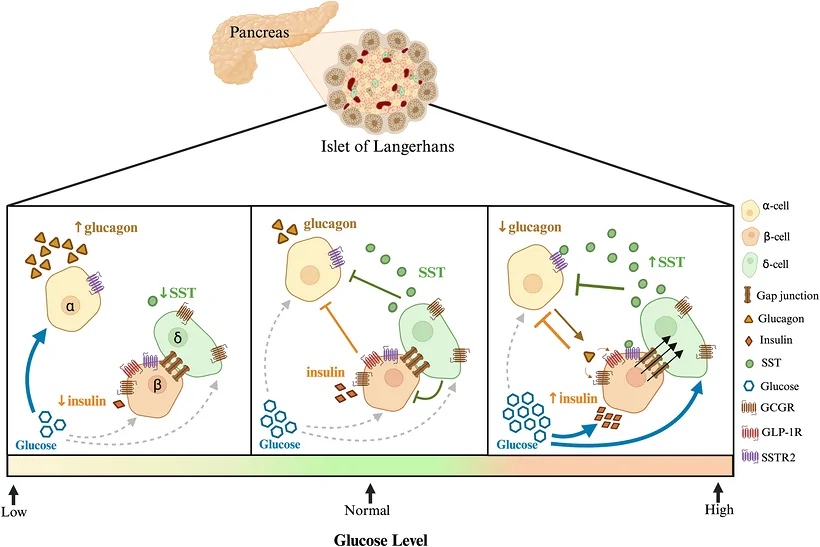
Glucose‐dependent regulation of glucagon, insulin, and somatostatin secretion and intra‐islet paracrine crosstalk between α‐, β‐, and δ‐cells across different physiological glucose states.  Islets found within the pancreas are composed of α‐, β‐, and δ‐cells that secrete endocrine hormones (glucagon, insulin, and somatostatin) to control blood glucose (Top). During hypoglycemia (<70 mg/dL; bottom left panel), low glucose stimulates glucagon secretion from pancreatic α‐cells; however, since glucose levels are below the threshold for the activation of β‐ and δ‐cell action potentials, both insulin and somatostatin (SST) secretion remain at basal levels. Their reduced inhibitory tone may further contribute to glucagon secretion. As blood glucose reaches euglycemia (∼70−120 mg/dL; bottom middle panel), α‐, β‐, and δ‐cells maintain basal levels of glucagon, insulin, and somatostatin to offset fluctuations in blood glucose levels while also preserving a balanced intra‐islet signaling. During the postprandial period when glucose levels rise (>120 mg/dL; bottom right panel), the β‐cells depolarize in a glucose‐dependent fashion, which then propagates to neighboring δ‐cells through gap junctions, promoting glucose‐dependent somatostatin secretion. Once secreted, somatostatin imposes an inhibitory brake on both α‐ and β‐cells via somatostatin receptor subtype 2 (SSTR2) to restrict glucagon and insulin secretion, respectively. While uncertainties remain about glucagon output under hyperglycemia, several in vitro studies on human islets have shown that residual glucagon pulsates from α‐cells and may act through β‐cell‐specific G‐coupled glucagon (GCGR) and/or glucagon‐like peptide 1 receptors (GLP‐1R), providing a feed‐forward signal that may amplify insulin secretion.

The endocrine tissue of the pancreas consists of microclusters of endocrine cells known as islets of Langerhans. The islets are composed of several endocrine cell types, including α‐cells, β‐cells, and δ‐cells, that function as an interconnected network to maintain blood glucose homeostasis (Figure 1) [1, 2]. Based on recent continuous glucose monitoring studies in healthy individuals without diabetes [3, 4], the normal glycemic range is between 70 and 140 mg/dL, but large carbohydrate‐rich meals, intensive exercise, and other factors are associated with elevations or reductions outside of this range from time to time. Glucagon, a counterregulatory peptide hormone released by α‐cells, acts on the liver to increase hepatic glucose output during hypoglycemia, exercise, or with prolonged fasting [5]. While α‐cell glucagon output is mainly dependent on circulating blood glucose levels, increasing evidence suggests that intra‐islet paracrine signaling from β‐ and δ‐cells could also regulate glucagon secretion. In short, insulin secretion from β‐cells and somatostatin (SST) secretion from δ‐cells suppress α‐cell activity when ambient glucose levels are high. During hypoglycemia (i.e., defined as blood glucose levels of <70 mg/dL), reduced inhibitory tone from pancreatic β‐ and δ‐cells stimulates α‐cells to secrete glucagon (Figure 1, left) [6, 7, 8, 9, 10]. Notably, even under low glucose levels, δ‐cells provide a constant (tonic) brake on α‐cells to prevent α‐cells from secreting glucagon in excess. Ultimately, this integrated intra‐islet network contributes to regulation of the glucagon counterregulatory response, which acts as a protective barrier against hypoglycemia.

**FIGURE 2:**
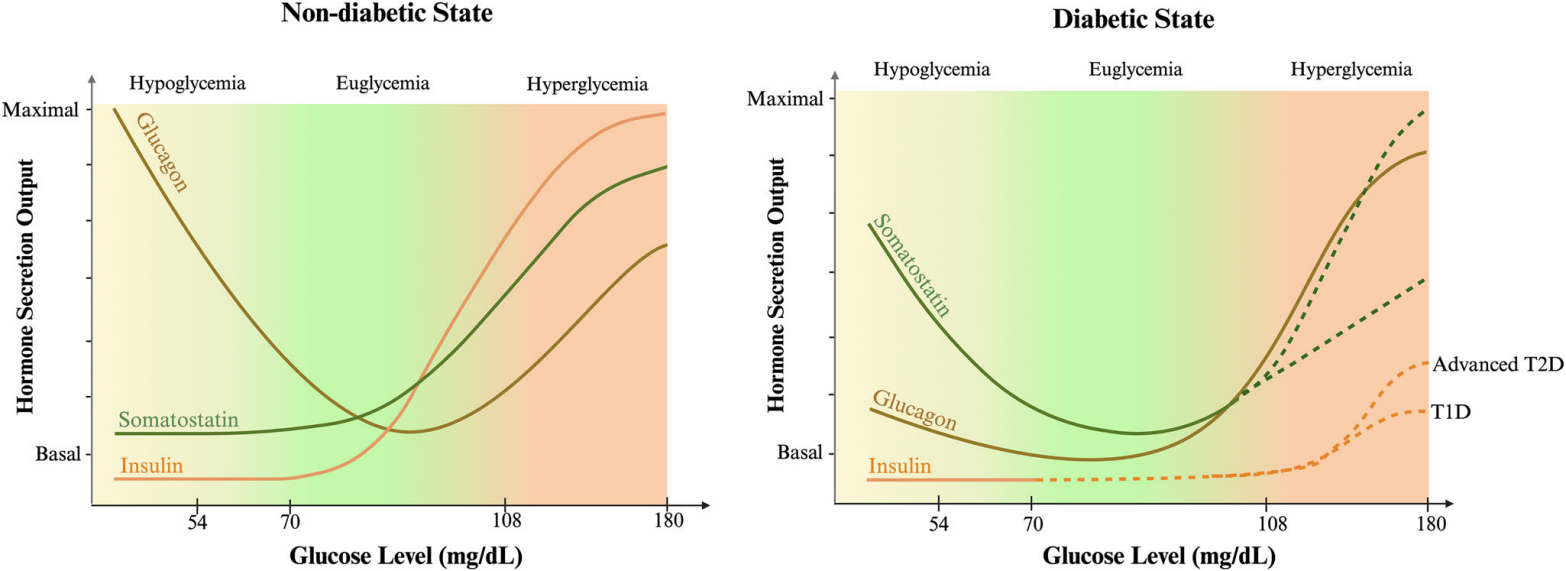
Glucagon, insulin, and somatostatin secretion across different levels of glycemia in nondiabetic (left) and diabetic (right) states. In a nondiabetic state (right), glucagon secretion is maximally stimulated, while insulin and somatostatin (SST) levels remain near basal levels during level 2 hypoglycemia (<54 mg/dL; pale yellow shading). Within the euglycemic range (light green shading), glucagon secretion normally declines and insulin and somatostatin secretion begin to rise. During hyperglycemia (orange shading), insulin and somatostatin rise dramatically in a glucose‐dependent manner. Notably, glucagon has been suggested to exhibit a reverse J‐shape response, where levels may decline at first but eventually increase to amplify insulin secretion via G‐coupled glucagon (GCGR) or glucagon‐like peptide receptors (GLP‐1R) signaling. In a diabetic state (right), however, the regulation of hormones is changed mainly due to alterations in intra‐islet crosstalk and architecture. During hypoglycemia, increased inhibitory tone from δ‐cells prevents glucagon secretion. At hyperglycemia, the autoimmune attack in T1D, or exhaustion of pancreatic β‐cells in advanced T2D, blunts endogenous insulin secretion. The loss of functional β‐cell mass may be a factor contributing to chronic hyperglucagonemia. As for δ‐cells, it remains unclear how somatostatin secretion changes in T1D or in advanced T2D. Some studies show that somatostatin may increase as a compensatory mechanism to regulate excess glucagon secretion. Other studies demonstrate that the underlying cause of glucagon hypersecretion during hyperglycemia could be due to reductions in inhibitory tone from δ‐cells. The figure was adapted and redesigned based on the conceptual framework illustrated previously by Huising et al. [67].

In type 1 diabetes (T1D) and late‐stage type 2 diabetes (T2D), glucagon counterregulation to hypoglycemia and to prolonged exercise is found to be impaired [11, 12]. It has long been proposed that the loss of functioning islet β‐cells and diminished endogenous insulin secretion increase α‐cell activity, leading to excess postprandial glucagon secretion in both types of diabetes (Figure 2) [13]. However, differences in the mechanisms for dysregulated glucagon likely exist since islet architecture differs between T1D and late‐stage T2D [1, 14]. To ameliorate hyperglucagonemia and achieve optimal glycemic control, individuals with T1D or late‐stage T2D often rely on insulin analogues and/or insulin secretagogues that frequently result in increased hypoglycemic exposure soon after the therapy is introduced. This, coupled with impaired glucagon counterregulation, which appears to worsen over time, further exacerbates hypoglycemia in individuals with T1D or late‐stage T2D [11]. To help manage insulin‐induced hypoglycemia or prolonged exercise, individuals on insulin therapy are left with having to increase carbohydrate feeding and/or relax exogenous insulin delivery [15].

**FIGURE 3:**
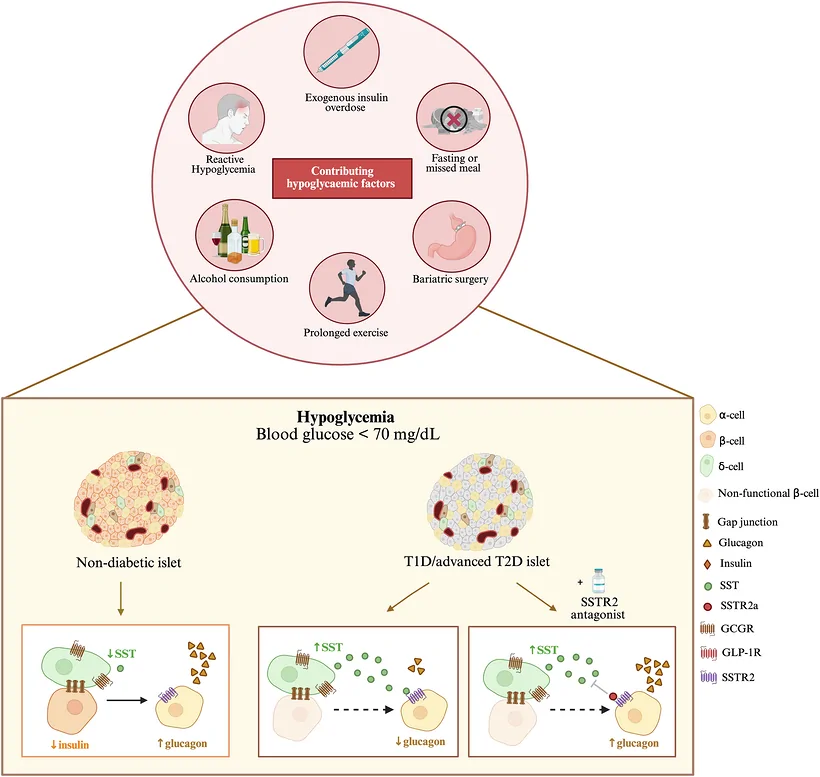
Common risk factors for hypoglycemia (top) and glucagon counterregulatory responses (bottom) in nondiabetic versus diabetic islets. Hypoglycemia can arise from exogenous insulin overdose, fasting or missed meals, reactive hypoglycemia, alcohol consumption, prolonged exercise, and complications of bariatric surgery. In nondiabetic islets, low glucose stimulates glucagon secretion from α‐cells, preventing blood glucose levels from dropping dangerously low. Notably, pancreatic β‐ and δ‐cells remain electrically silent due to the lack of propagation of depolarization currents between neighboring β‐ and δ‐cells. In T1D or advanced stages of T2D, a loss of β‐cell mass may lead to pathological elevation in somatostatin (SST) secretion, which may contribute to glucagon counterregulatory failure through augmenting SSTR2‐mediated inhibition on α‐cells. Pharmacological blockade of SSTR2 using an SSTR2 antagonist (SSTR2a) can alleviate the increased SST inhibitory tone on α‐cells by blocking the endogenous action of elevated somatostatin levels, ultimately restoring glucagon counterregulation response under hypoglycemic conditions.

To date, several studies have focused on understanding defects in the glucagon counterregulation response during insulin‐induced hypoglycemia in animal models of diabetes. Findings from rodent studies suggest that impaired glucagon secretion during hypoglycemia in T1D and advanced stages of T2D could be attributed to elevated SST signaling via somatostatin receptor subtype 2 (SSTR2) on α‐cells [7, 16, 17]. These studies are hampered, however, by the inability to accurately measure pancreatic islet‐derived SST‐14 in vivo using commercial antibodies (see Section 1.3). Nonetheless, these preclinical studies have led to the development of novel somatostatin receptor 2 antagonists (SSTR2a) that may be effective in restoring glucagon response during insulin‐induced hypoglycemia (Figure 3). This review compares paracrine regulation of glucagon secretion in nondiabetic and diabetic states. It also summarizes major findings from ex vivo and preclinical in vivo studies that investigated the potential effects of SSTR2 antagonism in restoring glucagon counterregulation during hypoglycemia in T1D or late‐stage T2D.

## 2 Cellular Organization and Interspecies Differences in Pancreatic Islet Architecture

The spatial arrangement of α‐, β‐, and δ‐cells within the islet plays an important role in regulating hormone output from endocrine cells of pancreatic islets. In rodents, pancreatic islets are composed of a β‐cell core with α‐ and δ‐cells residing in the periphery [18, 19]. In this arrangement, the majority of β‐cells are in direct contact with one another, allowing for synchronous Ca^2+^ responses within β‐cells during glucose stimulation [19]. In contrast to islet architecture in rodents, human islets exhibit a more mosaic pattern, in which α‐, β‐, and δ‐cells are reported to intermingle with one another [19, 20]. This cytoarchitecture arrangement of endocrine cells enables effective diffusion of paracrine factors through cell contacts. In addition to species‐specific architectural differences, islets themselves exhibit substantial heterogeneity in endocrine cell composition. In rodents, α‐ and β‐cells each constitute 15%–20% and 60%–80% of the total islet mass, respectively. Whereas, in human islets, the proportion of α‐ and β‐cells changes to 40% and 50% [19, 20]. In both rodent and human islets, δ‐cells’ filopodia‐like projections not only compensate for their scarcity within islets but also facilitate paracrine communication by forming contacts with neighboring α‐ and β‐cells [21].

Although the cellular composition of human islets has been well‐characterized, recent studies on human islets have revealed that islets exhibit substantial inter‐islet heterogeneity. Recently, 3D imaging of the human pancreas has shown that ∼50% of human islets are small insulin‐positive islets that are devoid of any α‐cells [22, 23]. The observed inter‐islet heterogeneity in cellular composition of human islets raises the possibility that β‐cell function may differ between α‐cell‐rich and α‐cell‐poor islets. Given the established role of glucagon in amplifying β‐cell activity through the glucagon and glucagon‐like peptide‐1 (GLP‐1) receptor signaling pathway [24, 25, 26], it is possible that local differences in α‐cell abundance and, therefore, glucagon exposure contribute to functional heterogeneity in β‐cells under glucose‐stimulated conditions [27]. However, direct functional comparisons of β‐cell secretory responsiveness between α‐cell‐rich and α‐cell‐poor islets have not been experimentally established yet.

Beyond inter‐islet heterogeneity, islets exhibit heterogeneity in β‐cell to β‐cell connectivity, with distinct β‐cell subpopulations contributing differently to insulin secretion and Ca^2^^+^ signaling [27]. Recent transcriptome analysis of islets has identified β‐cell subpopulations, including hubs, first responders, and leader cells, that coordinate insulin secretion and islet Ca^2^^+^ oscillations [27, 28]. The emergence of functional subtypes within islets may be influenced by local microenvironmental factors, including proximity to α‐ and δ‐cells and their exposure to paracrine signaling factors. Nevertheless, such heterogeneity may in turn influence the strength and dynamics of glucagon regulation across individual islets under varying glucose levels.

Islet vasculature may also play an important role in facilitating paracrine crosstalk across the islets. Early studies have speculated that blood vessels in islets possess a glomerulus‐like structure that facilitates crosstalk between different cell types [29]. Vascular imaging of rat pancreatic tissue has shown that blood vessels in rat islets follow a core‐to‐periphery arrangement, which may facilitate distribution of β‐cell derived hormones and paracrine factors that may influence the islets periphery [30]. Other studies have proposed a periphery‐to‐core and pole‐to‐pole arrangement of islet vasculature [31, 32]. However, given the substantial heterogeneity in islet architecture, it is plausible that different structural arrangements are associated with distinct patterns of vascular organization. In contrast to directional vascularization observed in rodent islets, human islets have been reported to contain a less structured and lower blood vessel density per islet area than in rodent islets (i.e., mouse islets) [20]. Three‐dimensional modeling of human islet vasculature has shown the localization of large capillaries around islet clusters that branch into grooves within individual islets. These large central vessels further branch out into smaller capillaries across the islet [33]. Although our understanding of human islet vascularization continues to evolve, evidence from both human and rodent islets suggests that the islet vasculature plays a critical role in regulating intra‐islet paracrine communication.

## 3 Neuroendocrine Regulation of Pancreatic α‐Cells

Pancreatic islets in both rodents and humans are innervated by the parasympathetic and sympathetic nervous systems. In rodent islets, parasympathetic nerves innervate both α‐ and β‐cells, coordinating insulin secretion during hyperglycemia [34]. On the other hand, sympathetic nerves densely innervate mouse islets and, in particular, are mostly associated with the α‐cells [35]. During hypoglycemia or fasting, sympathetic activation stimulates release of norepinephrine from the adrenal medulla, which will interact with α‐cell β_2_‐adrenergic receptors to stimulate glucagon secretion by facilitating Ca^2^^+^ through L‐type Ca^2^^+^ channels [36, 37]. During the cephalic and postprandial phase, parasympathetic pathways release acetylcholine, which acts on muscarinic receptors to trigger insulin and glucagon secretion [38]. Notably, the parasympathetic secretion of glucagon may be overridden during hyperglycemia as nutrient and intra‐islet paracrine inhibitors, including insulin and somatostatin, exert potent inhibitory effects on α‐cells, ensuring effective suppression of glucagon. Unlike rodent islets, nerves innervating human islets have been speculated to lack direct contact with endocrine cells. Specifically, autonomic nerves are found to be closely associated with blood vessels, thereby regulating hormone output by altering blood vessel tone [35].

## 4 Intrinsic and Paracrine Regulation of Glucagon Secretion

Similar to β‐ and δ‐cells, pancreatic α‐cells are electrically excitable [39, 40, 41]. This electrophysiological property allows α‐cells to intrinsically regulate their hormone secretion in response to changes in ambient glucose levels. Even during mild (level 1) hypoglycemia (i.e., glucose 54–70 mg/dL; 3.0–3.8 mM), α‐cells maintain a high electrical activity to facilitate Ca^2^^+^ influx via voltage‐gated calcium channels (mainly P/Q Ca^2+^ channels) and potentiate exocytosis of glucagon‐containing vesicles [39, 42, 43, 44]. Secreted glucagon, which appears to be maximally secreted in humans when glucose falls below 36 mg/dL (<2.0 mM) [45] (Figure 2, left panel), enters the hepatic portal vein directly and almost immediately increases hepatic glucose output by facilitating glycogenolysis while suppressing glycolysis and glycogenesis [5]. Since the liver clears glucagon after secretion, measuring glucagon in peripheral locations, such as during a blood draw, can underestimate glucagon release during hypoglycemia and exercise [46, 47]. As blood glucose rises back to the euglycemic range (defined as blood glucose of ∼70–108 mg/dL) [48], glucose metabolism within α‐cells promotes complete closure of ATP‐sensitive potassium (K_ATP_) channels, further depolarizing α‐cell membrane and ultimately reducing glucagon secretion to basal levels [39, 42, 44] (Figure 1, middle panel). While suppression of glucagon may be attributed to reduced intracellular Ca^2+^ levels at high glucose levels, this model has been challenged by studies showing high glucose levels inhibit glucagon secretion without reducing intracellular Ca^2+^ levels [49, 50]. These observations suggest that additional Ca^2+^‐independent mechanisms contribute to glucose‐mediated regulation of glucagon secretion.

Furthermore, the relationship between glucose levels and glucagon secretion does not follow a linear trend. This is because glucagon secretion is maximally inhibited in islets exposed to physiological glucose levels, with suppression becoming less pronounced at higher glucose levels and even increasing with pathological hyperglycemia (e.g., 360 mg/dL [20 mM] glucose) [41, 51]. Thus, glucagon shows a reverse J‐shaped response to glucose, increasing in response to hypo‐ and hyperglycemia (Figure 2, left panel). In vitro studies showed that the paradoxical increase in glucagon secretion during hyperglycemia may help stimulate insulin release in the late postprandial state when glucose is elevated [52, 85]. Overall, these findings illustrate that glucagon secretion from pancreatic α‐cells is precisely controlled by both glucose and paracrine factors from neighboring β‐ and δ‐cells via indirect and direct signaling pathways.

### 4.1 Role of β‐Cells in Regulating Glucagon Secretion

Insulin, an antagonist hormone to glucagon secretion, is released from pancreatic β‐cells in response to rising glucose levels (Figure 2, left panel). Insulin secretion is stimulated at 54 mg/dL (3.0 mM) glucose in human islets and at 90 mg/dL (5.0 mM) glucose in mouse islets [53, 54]. In both, secretion is half‐maximal at 180–216 mg/dL (10–12 mM) glucose and plateaus at glucose levels above ∼360 mg/dL (∼20 mM) [53]. Once secreted, insulin acts on the liver, skeletal muscle, and adipose tissue to facilitate glucose uptake and storage, primarily as glycogen. Within the pancreas, insulin has been shown to serve as a paracrine factor that suppresses glucagon secretion at high glucose levels (Figure 1, right panel) [13, 55]. This inhibitory effect was first observed in a hyperglycemic clamp study with nondiabetic human subjects, wherein a 46% reduction in plasma glucagon levels was observed following glucose‐insulin infusion [56]. Later ex vivo experiments involving static incubation of freshly isolated islets with an insulin receptor (IR) antagonist (i.e., S961) further elucidated these findings, as antagonizing IRs under high glucose reversed insulin‐mediated suppression of glucagon secretion [6, 8, 55]. Studies investigating the insulin mechanism of inhibition show that secreted insulin at high glucose triggers activation of α‐cell phosphodiesterase 3B (PDE3B), a cAMP‐hydrolyzing enzyme that suppresses glucagon secretion by reducing cAMP levels. Application of the PDE3B inhibitor cilostamide counteracted insulin's suppressive effect, leading to a 2.65‐fold increase in glucagon release under high‐glucose conditions [8].

Other in vitro experiments involving dispersed α‐cells showed that the lack of endogenous insulin within islets yields a V‐shaped response, with glucagon exocytosis being maximal under high glucose conditions [55]. Although dispersed α‐cells do not replicate the physiological islet microenvironment, these findings suggest that insulin‐mediated paracrine signaling is needed for suppression of glucagon secretion during hyperglycemia. Furthermore, the lack of insulin regulation could lead to changes in glucagon secretion pattern, diverging from the canonical J‐shape observed previously in intact islets.

Insulin maintains its suppressive effect on α‐cell glucagon release under euglycemic conditions. This is evident from euglycemic clamp studies on fasted nondiabetic and type 1 diabetic human subjects, in which glucagon secretion declined following intravenous insulin‐glucose infusion [56, 57]. Complementary findings from ex vivo studies have also shown that reversing insulin inhibition using S961 in both isolated mice and human islets boosts glucagon secretion near physiological glucose levels (i.e., ∼70 mg/dL [∼3.9 mM]) [58]. Although the exact inhibitory mechanism of insulin on α‐cell secretion during euglycemia remains unknown, existing data from experiments on isolated islets suggest that insulin‐induced inhibition of glucagon might be mediated via indirect signaling pathways, possibly involving SST secretion from pancreatic δ‐cells [58, 59, 60]. In RIP‐Cre^+/−^ ChR2‐YFP^+/−^ mice, optoactivation of β‐cells was shown to stimulate SST release while causing a 26% reduction in glucagon secretion from islets. d‐cell‐driven SST secretion was shown to be modulated by electrical currents that propagate from β‐cells to δ‐cells via gap junctions, as application of a gap junction inhibitor, carbenoxolone, abolished these currents. Furthermore, treatment with CYN 154806, a SSTR2a, relieved glucagon suppression following β‐cell optoactivation, suggesting glucagon suppression by β‐cells is mediated through δ‐cell activation [59].

Other studies have also implicated insulin in directly stimulating glucagon secretion via a switch‐on/switch‐off mechanism to help explain how insulin levels might regulate glucagon secretion [61, 62]. In brief, the glucagon switch‐on/switch‐off hypothesis proposes that a rapid decrease in insulin (the switch‐off) from pancreatic β‐cells is necessary to trigger glucagon secretion from the neighboring α‐cells during hypoglycemia [63]. It also suggests that the lack of this switch‐off signal, due to β‐cell loss in T1D, causes the impaired glucagon response that is characteristic of this disease state after just a few months of clinical disease onset (see Section 2). In addition, when glucose is elevated and insulin is switched on, this may prime a‐cells to be more prepared to secrete glucagon when glucose levels fall. This hypothesis is supported by an islet perfusion study on nondiabetic Wistar rats, where stimulating insulin secretion under high glucose concentration (i.e., insulin switch‐on) caused a more robust glucagon response upon glucose withdrawal compared to those islets incubated with glucose levels below the stimulatory threshold of insulin secretion [62]. Furthermore, an incremental reduction in intra‐islet insulin levels (i.e., insulin switch‐off) has been shown to serve as a stimulatory signal for α‐cells to release glucagon during hypoglycemia [61]. Aside from dynamic changes to intra‐islet insulin levels, expression of functional IRs in α‐cells is also crucial for glucagon stimulation during hypoglycemia [64, 65]. This is evident from in vivo studies wherein fasting‐induced hypoglycemia increased plasma glucagon levels in control mice, but not in α‐cell‐specific insulin receptor knockout mice (αIKO) [64]. Ex vivo experiments on islets with α‐cell–specific IR mRNA knockdown also show blunted glucagon secretion in response to low glucose conditions compared to those islets from control littermates [65]. Overall, these findings suggest that glucose‐induced insulin secretion prevents glucagon release at high glucose levels and that reductions in insulin levels provide a permissive signal for α‐cells to secrete glucagon at low glucose (Figures 1 and 2).

### 4.2 Contribution of Other β‐Cell Paracrine Factors to Glucagon Regulation

Beyond insulin and SST, other intra‐islet paracrine factors contribute to regulation of glucagon secretion [25, 66, 67].  γ‐aminobutyric acid (GABA), an inhibitory neurotransmitter released by pancreatic β‐cells, is cosecreted with insulin during hyperglycemia. Secreted GABA suppresses glucagon secretion by facilitating Cl^−^ influx and α‐cell membrane hyperpolarization [69]. Serotonin, another inhibitory neurotransmitter, has emerged as a potential modulator of pancreatic islet hormone secretion. In adult mice, average serotonin production has been reported to be under the detection limit of available assays. To date, several studies in rodent islets have illustrated an increase in serotonin levels specifically under conditions of high metabolic demand (i.e., pregnancy or a high‐fat diet), where serotonin exerts autocrine feedback to promote β‐cell proliferation [70, 71]. On the other hand, studies involving human islets have shown that β‐cells express a higher level of serotonin [72] than rodents, suggesting a physiological role for this neurotransmitter in pancreatic hormone regulation. In healthy human islets, serotonin has been shown to directly attenuate glucagon secretion during hyperglycemia by lowering cAMP levels [72]. Lastly, urocortin‐3 (UCN3), another intra‐islet factor released by pancreatic β‐cells during hyperglycemia, has been shown to suppress glucagon secretion in isolated islets indirectly by promoting secretion of the inhibitory peptide SST [73, 74]. Similar to GABA and serotonin, UCN3 is cosecreted with insulin from β‐cells during hyperglycemia. Once secreted, UCN3 interacts with corticotropin‐releasing factor receptor‐2 on δ‐cells to promote SST secretion, which negatively feeds‐back on α‐ and β‐cells to prevent hormone hypersecretion (see Section 1.5) [74, 75]. Collectively, these β‐cell factors contribute to suppression of glucagon secretion during hyperglycemia via direct and indirect mechanisms.

### 4.3 Regulation of Glucagon Secretion by δ‐Cells

SST is a cyclic peptide that exists in two distinct biological isoforms, SST‐14 and SST‐28. SST‐14 is 14 amino acids long and predominantly secreted by pancreatic δ‐cells. On the other hand, SST‐28 is 28 amino acids long and mainly released by enteroendocrine D cells of the gastrointestinal (GI) tract [76]. Despite their differences, both isoforms play important roles in regulating hormone secretion from exocrine and endocrine tissues. In the pancreas, SST‐14 acts as a local paracrine inhibitor of insulin and glucagon secretion (Figure 1) [2, 77]. Upon secretion, SST‐14 activates the inhibitory subunit of G protein (G_i_) coupled to the somatostatin receptors (SSTR) on α‐ and β‐cells. Activated G_i_ inhibits cAMP production and Ca^2+^ influx, leading to suppression of hormone secretion by pancreatic α‐ and β‐cells [78]. Although the SST‐14‐mediated mechanism of glucagon suppression is conserved between species, the distribution of SSTR subtypes in islet cells varies. For instance, in rodent pancreatic islets, α‐cells and β‐cells predominantly express SSTR2 and SSTR3, respectively [78, 79]. While in humans, SSTR2 is found to be the dominant receptor subtype that is expressed by both pancreatic α‐ and β‐cells [78, 80]. Despite the presence of SST‐14 in circulating blood, quantifying plasma SST‐14 levels presents several challenges. This is because circulating SST‐14 has a very short half‐life (<2 min) due to its susceptibility to proteolytic degradation, which results in plasma levels that are below the detection limit of available quantitative assays [77, 81]. Moreover, commercial assays do not distinguish between SST‐14 and SST‐28. Given these challenges, most of the work centering around quantifying endogenous SST‐14 and its paracrine role in glucagon regulation levels has been done ex vivo using isolated islets or pancreas perfusion [81].

SST‐14 is secreted from pancreatic α‐cells tonically and at glucose levels as low as 54 mg/dL (3 mM), thereby providing a constant inhibitory effect on α‐cells even under low glucose conditions. This is supported by in vivo studies wherein SSTR2 antagonism in rodents undergoing insulin‐induced hypoglycemia increases plasma glucagon levels compared to vehicle‐treated controls [82]. Similar to insulin, SST secretion increases in a dose‐dependent manner with glucose, likely to help limit insulin and glucagon hypersecretion (Figure 2, left panel) [41, 51, 68]. Thus, under most physiologic conditions, secreted SST‐14 prevents hyperglucagonemia by acting as a biological brake on α‐cell hormone secretion (Figure 1, right panel). This effect was shown in an in vivo study wherein global ablation of SSTR2 in high fat diet (HFD)‐fed mice led to an increase in nonfasting plasma glucagon levels and a decrease in liver glycogen content compared to wild‐type (WT) HFD‐fed mice [83]. Ex vivo studies have also shown a significant increase in glucagon secretion in nondiabetic islets treated with SSTR2 compared to vehicle‐treated group, suggesting that SST mediates inhibition of α‐cells in a paracrine manner at high glucose levels [6, 55, 84]. Thus, under low glucose conditions, basal SST levels contribute to tonic inhibition of glucagon secretion. This effect is evident from hyperinsulinemia‐hypoglycemic clamp studies on nondiabetic rats, in which SSTR2a infusion (i.e., PRL‐2903) led to a 3.3‐fold increase in plasma glucagon levels. Additionally, nondiabetic rats treated with SSTR2a required more insulin infusion to stay within the target hypoglycemic range, suggesting that reversal of SST‐mediated inhibition elevates glucagon levels, which may shift the glycemic set point upward [82]. Ex vivo experiments on isolated or perfused pancreatic islets also yielded results consistent with in vivo studies. In pancreatic slice perfusion studies, the addition of PRL‐2903 to a low glucose perfusion medium resulted in a 2.4‐fold increase in glucagon secretion compared to the glucagon secretion from slices exposed only to low glucose levels [82]. Overall, current knowledge suggests that islet‐derived SST plays an important role in regulating α‐cell activity and that reduced inhibitory signals, rather than complete absence, allow for glucagon secretion under a low glucose environment (Figure 1).

### 4.4 Regulation of Glucagon by Gut‐Derived Hormones

In addition to cell−cell communication within pancreatic islets, gut hormones including glucagon‐like peptide‐1 GLP‐1, glucose‐dependent insulinotropic peptide (GIP), and ghrelin all appear to contribute to the regulation of glucagon secretion and glucose homeostasis [24]. GLP‐1 is an incretin hormone that is released by enteroendocrine L cells upon feeding [25]. GLP‐1 has an insulinotropic effect on pancreatic β‐cells through activation of a G‐protein‐coupled receptor (GPCR) signaling pathway that is mediated by enhanced intracellular cAMP levels [24]. In α‐cells, GLP‐1‐induced cAMP signaling suppresses glucagon secretion. In vitro studies on human islets show that the suppressing effect of GLP‐1 on glucagon secretion is mediated directly through inhibition of α‐cell P/Q Ca^2^^+^ channels rather than via high glucose stimulation of insulin or SST [86]. In rodents, GLP‐1 has been shown to inhibit glucagon secretion via stimulation of SST, as antagonizing SSTR2 in the presence of exogenous GLP‐1 augmented glucagon secretion compared to exogenous GLP‐1 treatment alone [132]. Although characterizing GLP‐1R expression in pancreatic islets across species (including humans) remains technically challenging, interspecies differences in islet GLP‐1R expression may contribute to variability in reported findings.

GIP is another incretin hormone released by enteroendocrine K cells during hyperglycemia [137]. GIP has been shown to increase glucagon release from α‐cells under both low and high glucose levels [87]. Notably, the glucagon‐stimulating effect of GIP is reported to be more pronounced when certain amino acids (e.g., arginine, alanine, glutamine) are present [88]. Under low glucose levels, GIP‐induced glucagon secretion from α‐cells helps prevents hypoglycemia through GPCR signaling [24, 84, 85, 87]. Under high glucose levels, GIP acts as an insulin secretagogue by modulating α‐to‐β cell communication [24, 88]. In vivo studies have demonstrated that GIP gene knockout impairs glucose tolerance in mice, highlighting GIP's crucial role in maintaining glucose homeostasis largely via insulin secretion [88]. Although GIP and GLP‐1 are both secreted in response to nutrient stimuli, the relative contribution of their opposing effects on glucagon secretion remains unclear. Nevertheless, both incretins serve an important physiological function in maintaining glucose homeostasis.

Ghrelin, a gut‐derived hormone known as the “hunger hormone,” is released by epsilon cells of the stomach before meals. This hormone acts to increase glucose levels by promoting glucagon secretion and inhibiting insulin release [89]. The glucagonotropic effect of ghrelin has been shown to be mediated via direct signaling within α‐cells [90]. The insulin‐inhibiting effect of ghrelin on insulin secretion has been shown to be mediated by δ‐cells, where the local release of SST via ghrelin helps to provide negative feedback on β‐cell insulin secretion [91, 92]. Although ghrelin stimulates SST secretion, the inhibitory effects of SST on glucagon secretion may be overridden during fasting or hypoglycemia. Collectively, the gut‐derived hormones contribute to regulation of glucagon secretion across varying glucose levels through both direct and indirect mechanisms.

## 5 Altered Regulation of Glucagon Secretion in Diabetes

Diabetes mellitus is a multihormonal disease characterized by chronically elevated blood glucose levels. T1D is characterized by a lack of endogenous insulin secretion due to an autoimmune attack of β‐cells [93], while T2D is a slower progressive disease with respect to the development of hyperglycemia, where β‐cell insulin secretion eventually fails to compensate for insulin resistance in the liver, muscle, and adipose tissue [93,94,134]. Recently, digital scanning of pancreatic tissue slide sections and 3D imaging of whole human pancreas has shown that in the progression of T1D, autoimmune attack on β‐cells occurs in a heterogeneous manner, with small clusters of β‐cells being disproportionately affected and residual β‐cells persisting in larger islets [22,95]. It has been suggested that small islets contain a greater insulin content and, therefore, a higher pool of insulin antigen, potentially rendering them more susceptible to autoimmune attack [95]. Another possible mechanism protecting larger islets from autoimmune destruction in T1D may be the presence of α‐cells. Under metabolic stress, α‐cells upregulate prohormone convertase 1/3, which allows for local production of GLP‐1 during proglucagon processing [97, 98, 99]. Notably, intra‐islet GLP‐1 production may be part of a system that facilitates β‐cell survival and proliferation [99], therefore, offering a protective barrier against autoimmune attack of β‐cells in the early stages of T1D development.

In early stages of T2D, peripheral insulin resistance increases metabolic demand on β‐cells by causing insulin hypersecretion to restore normoglycemia. During this phase, β‐cells undergo hypertrophy and hyperplasia to sustain elevated insulin needs [,^94^, ^101^]. As metabolic stress persists in T2D, chronic hyperglycemia and inflammation induce β‐cell dedifferentiation. During this stage, the transcription of critical glucose‐sensing factors of β‐cells, such as GLUT2 and glucokinase, is downregulated [93, 101]. In some cases, β‐cells have been found to undergo transdifferentiation into α‐cells, which may further exacerbate hyperglycemia. During the late stage of T2D progression, β‐cell mass is profoundly reduced due to β‐cell exhaustion and apoptosis [93, 102]. In addition to changes in α‐ and β‐cell populations, changes to islet size distribution have also been observed in T2D donors. Immunohistochemical examination of T2D human islets revealed a preferential loss of large islets in islets from T2D donors [104]. This is in contrast to the observations made from the pancreatic tissue examination of T1D donors, where small islets were reported to be more susceptible to immune cell attack [95]. Although these studies offer new perspectives on the morphological changes of pancreatic islets during T2D progression, limitations remain in drawing conclusions from two‐dimensional histological examinations. Future studies may benefit from more robust experimental approaches to further refine changes in islet morphology during diabetes progression at the whole‐pancreas level.

### 5.1 Intrinsic α‐Cell Defects in Diabetes

In both T1D and T2D, glucagon secretion is typically dysregulated, with excessive secretion during fasting and hyperglycemia as well as inadequate secretion during hypoglycemia (Figures 2 and 3) [93, 105]. The worsening of hyperglycemia because of glucagon could contribute to several adverse health outcomes, such as neuropathy, nephropathy, and retinopathy [93]. At the other extreme, impaired glucagon counterregulatory response eliminates the body's natural first line of defense against hypoglycemia [11, 106]. Over time, repeated episodes of hypoglycemia blunt hormonal and sympathetic responses, marking the onset of hypoglycemia‐associated autonomic failure (HAAF). Development of HAAF in diabetes is life‐threatening, as patients fail to recognize or respond to falling glucose levels, allowing progression to severe neuroglycopenia with symptoms ranging from dizziness, confusion, seizures, to coma, or even death (if untreated) [107, 108]. To date, several studies have focused on understanding how α‐cell intrinsic defects contribute to the dysregulated glucagon secretion in diabetes. A recent study by Gao et al. has shown that K_ATP_ channels activity in α‐cells may be altered in T1D [109]. This is supported by experiments demonstrating that treatment with a low dose of tolbutamide enhances glucagon secretion in islets isolated from T1D donors under low glucose levels, while it attenuates glucagon secretion in nondiabetic controls. These findings suggest that α‐cell K_ATP_ channel activity resembles that of β‐cells in T1D, with K_ATP_ channels possibly remaining pathologically open under low glucose conditions. Consequently, sulfonylurea‐induced K_ATP_ channel closure may promote α‐cell activation, restoring glucagon counterregulatory response under low glucose conditions [109]. Previously, Huang et al. have shown that α‐cells in streptozocin (STZ)‐induced mice exhibit increased action potential amplitude and frequency, indicating enhanced cellular excitability compared with control islets. Additionally, α‐cells from STZ‐treated mice were found to contain larger glucagon granules and increased glucagon exocytosis compared to those of control littermates [110]. Notably, increased glucagon granule exocytosis was independent of changes in Ca^2^^+^ influx, as whole‐islet Ca^2^^+^ recordings did not reveal differences between STZ‐treated and control mice [110]. Collectively, these results indicate that enhanced glucagon secretion in this model of T1D is not driven by Ca^2^^+^ but rather by changes in electrophysiological properties of α‐cells.

Studies on T2D islets have also shown alterations in ion channel behavior in α‐cells that may contribute to reductions in α‐cell responsiveness at low glucose levels. In T2D islets, P/Q Ca^2^^+^ channel activity has been shown to decrease under low glucose conditions but increase at high glucose levels [111]. Further patch‐seq analyses have shown that poor glucagon exocytosis under low glucose conditions does not correlate with reduced channel expression. Furthermore, α‐cells from T2D donors were found to be enriched in developmental and endocrine lineage‐associated transcripts, suggesting that α‐cells may adapt to a less mature phenotype. Notably, α‐cells from mice fed a high‐fat diet appear to acquire β‐cell‐like electrophysiological properties, characterized by shifts in Na^+^ channel inactivation and P/Q Ca^2^^+^ channel uncoupling from exocytosis [111, 112]. Collectively, these results illustrate that under the diabetic state, intrinsic defects within α‐cells likely contribute to dysregulated glucagon secretion during both hypo‐ and hyperglycemia.

Counter to the argument that the restoration of α‐cell function (or glucagon receptor agonists) might be a desirable therapeutic target for people living with T1D or T2D are the striking preclinical data that removal of glucagon action or secretion can effectively restore functional β‐cell mass in type 1 diabetic mice and enhance the function of human islets under hyperglycemia in diabetes [113].

### 5.2 Defective β‐ to α‐Cell Crosstalk

In addition to intrinsic defects in α‐cell signaling contributing to defective glucagon secretion, interruptions in islet paracrine crosstalk have also been associated with hyperglycemia pathogenesis in diabetes. Hyperglucagonemia, a common complication of diabetes, is characterized by dysregulated glucagon secretion in both fasting and postprandial states. In T1D, hyperglucagonemia results from loss of pancreatic β‐cells and thus lack of endogenous insulin to suppress inappropriate glucagon secretion [138]. In T2D, it is manifested due to lack of α‐cell responsiveness to residual endogenous insulin secretion [13, 64, 115]. In both, impaired insulin‐mediated inhibition of glucagon secretion contributes to pathological elevations in circulating plasma glucagon levels and chronic hyperglycemia (Figure 2, right panel).

Beyond the disrupted insulin‐mediated suppression of glucagon during hyperglycemia, the lack of switch‐on and switch‐off signaling cues from β‐cells has been implicated with glucagon counterregulation failure in diabetes [9, 61]. This effect is illustrated in perfusion studies of STZ‐rat pancreata in which reduced insulin secretion at high glucose was associated with blunted glucagon secretion following glucose deprivation. Notably, restoring the insulin switch on‐and‐off signal by adding exogenous insulin and subsequently withdrawing insulin and glucose led to a ∼2.3‐fold increase in glucagon secretion compared to islets without this signaling restored [61]. The loss of the insulin switch‐on and switch‐off mechanism may also explain why insulin‐based therapies contribute to glucagon counterregulation failure in diabetes. In particular, elevations in circulating insulin levels owing to imperfect dosing of insulin analogues and secretagogues prevent α‐cells from sensing the insulin switch‐off signal that they normally depend on to release glucagon during hypoglycemia. Ultimately, this persistent switch‐on signal from elevated insulin levels could prevent glucagon secretion, which may further exacerbate hypoglycemia risk in individuals with diabetes.

### 5.3 Defects in δ‐Cell Mediated Glucagon Regulation

Over the past few decades, defective SST output from pancreatic δ‐cells has emerged as a key driver of impaired glucagon release in diabetes. Ex vivo and in situ pancreas perfusion studies showed that excessive glucagon secretion during hyperglycemia may result from reduced inhibitory tone from pancreatic δ‐cells under high glucose conditions (Figure 2, right panel). Notably, this defect could not be reversed by sustained exogenous SST treatment, as α‐cells appear to adopt an SST‐resistant phenotype [55, 114]. While these findings provide valuable insight into how metabolic stress (i.e., high‐fat feeding) could perturb SST secretion during hyperglycemia, further studies are needed to confirm these observations in animal models of T1D and advanced T2D.

Dysregulation of SST secretion is not limited to high‐glucose conditions but is also observed under low glucose states. Such an effect is evident from studies illustrating a pathological elevation in both islet SST content and secretion under low glucose environments (Figure 2) [7, 60]. Recently, in a seminal study, Hill et al. showed that electrically silent β‐cells clamp neighboring δ‐cells through gap junctions, thereby suppressing δ‐cell activity and SST release under low glucose conditions (Figure 2, left panel). In a T1D setting, however, downregulation of gap junctions due to the autoimmune destruction of β‐cells was shown to result in a loss of electrical coupling between neighboring δ‐ and β‐cells, contributing to elevated SST secretion (Figure 2, right panel) [60]. While it remains unclear if expression of gap junction proteins is downregulated in T2D, ex vivo studies on islets of Fhβ1KO mice—a murine model with dysfunctional β‐cells resembling that of T2D—have also shown a six‐fold increase in SST secretion correlating with a 75% reduction in glucagon secretion under very low glucose conditions (i.e., ∼18 mg/dL or 1 mM glucose). Reversal of SST‐mediated α‐cell suppression through the addition of CYN154806 led to a 143 ± 11% increase in glucagon secretion from Fhβ1KO islets compared to islets from control counterparts [7]. This observation indicates that elevations in SST secretion suppress glucagon secretion in T2D and that reversal of SST‐mediated inhibition using SSTR2a could restore glucagon counterregulatory response to hypoglycemia.

Consistent with observations made in an animal model of T1D, increased SST secretion was correlated with reduced glucagon secretion in islets from T1D human donors under low glucose conditions [60]. Although glucagon secretion was not stimulated at 18 mg/dL (1 mM) glucose alone, treatment of islets with CYN154806 led to a 68 ± 15% increase in glucagon secretion [60]. Similarly, human T2D islets exhibited blunted glucagon secretion in response to low glucose compared to their nondiabetic counterparts. However, reductions in glucagon levels could not be attributed to SST levels due to lack of statistical significance between T2D and nondiabetic groups [7]. Nevertheless, these findings support the concept that increased inhibitory input from δ‐cells via SST secretion might underlie the defects in glucagon counterregulation response to hypoglycemia in diabetes (Figures 2 and 3).

### 5.4 Alteration to Islet Vasculature and Innervation in Diabetes

Aside from alterations in cellular composition of islets and interruptions in intra‐islet communication, vasculature remodeling in islets further contributes to pathogenesis and progression of diabetes. Morphological studies on islets from T2D diabetic donors have shown that islet capillaries exhibit fragmentation and thickening of the basement membrane, which may impair nutrient delivery and gas exchange within the islet microenvironment [20, 116]. This may further exacerbate diabetes pathophysiology by causing local hypoxia and ultimately β‐cell dysfunction [20, 117]. In addition, alterations in islet vasculature may impact diffusion efficiency of intra‐islet factors, contributing to defective paracrine crosstalk and hyperglucagonemia discussed in Sections 2.1 and 2.2. With respect to islet vasculature remodeling in T1D, studies have yielded various findings. Granlund et al. documented an increase in islet vascular density and upregulation of angiogenetic factors in pancreatic biopsies from T1D donors [118]. While Richardson et al. reported preservation of vascular density despite significant β‐cell loss, suggesting that other mechanisms may account for dysregulated glucagon secretion in T1D [119]. Contrary to the findings of both of these studies, Wang et al. observed reductions in the density of islet microvessels in a mouse model of T1D [135]. The lack of consensus regarding islet vascular remodeling in T2D may be attributable to methodological differences between studies, including variations in imaging techniques, tissue processing, and approaches used to quantify vascular architecture.

In addition to changes in islet vasculature, autonomic nerves innervating islets undergo remodeling. During T1D onset, lymphatic infiltration contributes to sympathetic nerve loss in both rodent and human islets [135], which may underlie defects in α‐cell stimulation during hypoglycemia. As for T2D, tissue clearing and 3D imaging of the whole pancreas have revealed an increase in nerve density of both STZ‐treated mice and human T2D pancreata [136]. This increase in nerve density may be attributed to nerve regeneration and metabolic stress‐induced remodeling; however, the functional implications of these structural changes for α‐cell and β‐cell function remain incompletely understood.

### 5.5 Pharmacologic Modulation of Islet Cell Signaling in Diabetes

Current therapies for improving fasting plasma glucose in T1D and late‐stage T2D involve administration of long‐acting insulin analogues that provide basal insulin levels needed to suppress glucagon hypersecretion and prevent excessive hepatic glucose output. As for postprandial plasma glucose, the conventional treatment includes post‐meal bolus injection of short‐acting insulin analogues that mimic meal‐induced insulin spikes [120, 121]. In addition to exogenous insulin therapies, incretin‐based therapies such as GLP‐1 receptor agonists (GLP‐1‐RAs) are promising for treatment of hyperglycemia in individuals living with diabetes. GLP‐1‐RA was initially viewed as a treatment for T2D due to its ability to enhance insulin secretion through activation of the GLP‐1R signaling pathway in β‐cells [120]. However, recent studies suggest that GLP1‐RAs could also serve as an adjunctive treatment for individuals with T1D. GLP‐1RAs aid in reducing postprandial hyperglycemia by enhancing insulin secretion, mediating a glucagonostatic effect on pancreatic α‐cells, and possibly promoting β‐cell survival through stimulation of GLP‐1 signaling if given early on in the disease development [122, 123]. More recently, retatrutide, a triple glucose‐dependent agonist, has emerged as a groundbreaking treatment for obesity and T2D. This synthetic peptide has been shown to effectively lower blood glucose levels and promote weight loss by targeting GLP‐1, GIP, and glucagon receptors in clinical trials [124, 125]. However, it should also be noted that mice lacking non‐β‐cells (namely, mice with no α‐cells, δ‐cells, or γ‐cells) had normal blood glucose homeostasis, enhanced glucose tolerance, normal insulin sensitivity, and restricted body weight gain under a high‐fat diet [125]. These and other findings that pancreatic α‐cells appear to worsen glycemic control in mouse models of diabetes [126, 127] raise the question of the importance of intra‐islet signaling in health and disease.

While insulin analogues and secretagogues are effective in managing chronic hyperglycemia, they may also cause hypoglycemia as an unintended side effect, as described earlier. In parallel, pathological elevation in islet‐derived SST and its contribution to glucagon counterregulatory failure during hypoglycemia have shifted interest toward managing hypoglycemia risk in diabetes by targeting increased inhibitory tone by SST on glucagon secretion using SSTR2as. In short, this new class of drugs has been shown to compete with islet‐derived SST‐14 for binding to SSTR2, preventing endogenous SST from inhibiting glucagon secretion from pancreatic α‐cells [82] (Figure 3).

Preclinical studies on STZ‐injected rats using PRL‐2903 have shown an increase in plasma glucagon levels compared to the placebo group [17, 128]. More recently, ZT‐01, a novel SSTR2 antagonist, has been shown to be effective in ameliorating glucagon counterregulation in both T1D and T2D rats undergoing insulin‐induced hypoglycemia [16, 128]. In high‐dose STZ rats (a model of T1D), ZT‐01 delayed the onset of level 1 hypoglycemia (i.e., blood glucose of 3 mmol/L) by ∼65 min compared with PRL‐2903. In addition, this highly selective SSTR2 antagonist has superior pharmacokinetic properties, further supporting its potential therapeutic advantage [128]. In high‐fat‐fed, low‐dose STZ rats (a model of T2D), ZT‐01 also increases glucagon counterregulation during hypoglycemia [16] and may improve overall glucose metabolism [129]. Although the exact mechanism through which ZT‐01 improves glucose metabolism in T2D remains poorly understood, existing data from healthy mouse islets treated with SSTR2a suggest that this class of drug promotes glucagon secretion from α‐cells, which in turn enhances insulin release [85, 130]. Currently, ZT‐01 is in a clinical phase II trial for evaluating its effectiveness and safety in patients with and without T1D [81]. According to the recent data from a randomized crossover study, ZT‐01 administration in subjects with T1D led to a marked increase in plasma glucagon levels compared to the placebo group. In this trial, treatment‐related adverse effects were not clinically significant; however, an increase in circulating growth hormone has been observed in subjects receiving ZT‐01 treatment [131]. As for T2D, preclinical data from D'Souza et al. have demonstrated an increase in circulating C‐peptide levels in diabetic T2D rat models, suggesting that any glucagon‐mediated increase in hepatic glucose output may be partially offset by insulinotropic effects [16, 129]. Although this finding may suggest a therapeutic potential for ZT‐01 in improving overall glycemia and ameliorating nocturnal hypoglycemia, further research is needed to assess the risk of hyperglycemia associated with ZT‐01 in patients with T2D. Taken together, these findings suggest that targeting individual regulatory pathways may not be sufficient to restore counterregulatory glucagon response without avoiding unintended consequences. Therefore, emerging pharmacological approaches should aim to target multiple components of islet crosstalk to achieve optimal glucose control in diabetes.

## 6 Conclusion

Beyond glucose, intra‐islet paracrine signals from pancreatic β‐ and δ‐cells contribute to regulation of glucagon secretion from α‐cells. Under high glucose levels, increased insulin and SST secretion suppress α‐cell activity. As glucose levels fall, incremental decreases in insulin levels and reduced inhibitory tone from pancreatic δ‐cells provide a permissive signal for α‐cells to secrete glucagon. In T1D and late‐stage T2D, the intra‐islet paracrine network between these endocrine cells is typically compromised, leading to excess glucagon secretion at high glucose levels and impaired secretion under low glucose conditions. To date, several experimental studies have highlighted that defects in insulin switch‐on/switch‐off signaling and pathological elevations in the SST inhibitory tone may underlie the pathophysiology behind glucagon counterregulation failure in diabetes, yet significant gaps remain in fully understanding the mechanisms. Recently, selective SSTR2 antagonists have emerged as a promising therapeutic approach in alleviating insulin‐ or secretagogue‐induced hypoglycemia. Although these treatments offer a novel approach for hypoglycemia prevention, further study is needed to evaluate their long‐term effect on islet paracrine crosstalk and overall glycemia management in diabetes.

## Author Contributions

S.F.: Writing – original draft, writing – review and editing, J.V.R.: Writing – review and editing. M.C.R.: Writing – review and editing.

## Funding

S.F. has received a CIHR Canada Graduate Scholarship–Master's (CGS‐M). J.V.R. has funding support from NSERC (RGPIN‐2022‐04454) and CIHR (PJT‐204010).

## Conflicts of Interest

M.C.R. is a founder of Zucara Therapeutics and serves as a consultant for Eli Lilly, the Jaeb Centre for Health Research, and Zucara Therapeutics. J.V.R. and S.F. have no conflicts to disclose.
